# Evaluation of Models of Parkinson's Disease

**DOI:** 10.3389/fnins.2015.00503

**Published:** 2016-01-19

**Authors:** Shail A. Jagmag, Naveen Tripathi, Sunil D. Shukla, Sankar Maiti, Sukant Khurana

**Affiliations:** ^1^Department of Biology, Indian Institute of Science Education and ResearchKolkata, India; ^2^Department of Zoology, Government Meera Girl's CollegeUdaipur, India

**Keywords:** Parkinson's Disease, Parkinsonian Disorders, lewy bodies, neurodegeneration, ventral tegmental area (VTA), toxin models, genetic models, substantia nigra pars compacta (SNc)

## Abstract

Parkinson's disease is one of the most common neurodegenerative diseases. Animal models have contributed a large part to our understanding and therapeutics developed for treatment of PD. There are several more exhaustive reviews of literature that provide the initiated insights into the specific models; however a novel synthesis of the basic advantages and disadvantages of different models is much needed. Here we compare both neurotoxin based and genetic models while suggesting some novel avenues in PD modeling. We also highlight the problems faced and promises of all the mammalian models with the hope of providing a framework for comparison of various systems.

## Parkinson's disease—model utilization for therapeutics

Parkinson's disease (PD) is a common neurodegenerative disorder, with cardinal features of akinesia, bradykinesia, rigidity, and tremors (Rodriguez-Oroz et al., 2009). The neuropathological hallmarks of PD are the loss of dopaminergic neurons in the SubstantiaNigra pars compacta (SNc) and the formation of intra-neuronal proteinaceous inclusions, called Lewy Bodies (LBs). Loss of neurons from other brain regions has also been observed in the later stages of the disease, such as the cholinergic nucleus basalis of Meynert, many subnuclei in the thalamus and amygdala, and the serotoninergic neurons of the raphe nucleus (Jellinger, 1991; Braak et al., 2000, 2003). In most cases of PD, injury or environmental insult induced changes in the brain connectivity and gene expression, with genetic factors contributing to the predisposition, are suspected to cause the disease but in a smaller fraction of cases, between 10 and 20%, genes are known to be the culprits for causation. Genetic defects in mitochondrial function (Winklhofer and Haass, 2010), dysfunction of the ubiquitin-proteosome pathway (McNaught et al., 2001), and alterations of free radical formation (Palacino et al., 2004) have been shown to play a role in familial PD. Studies have reported increases in the sensitivity of mice with these defects to neurotoxins (Nieto et al., 2006; Haque et al., 2012).

The use of animals to model different aspects of PD phenotype allows us the ability to study both disease progression and explore possible treatments. While none of the currently available models of PD completely phenocopy the disease but they have contributed extensively to our knowledge of PD. So far experimental models have been of two major types: (A) Toxin models and (B) Genetic models. An understanding of different PD models can enhance the ability of PD researcher to employ appropriate models for their experiments.

## Toxin based models

**Figure 1:**
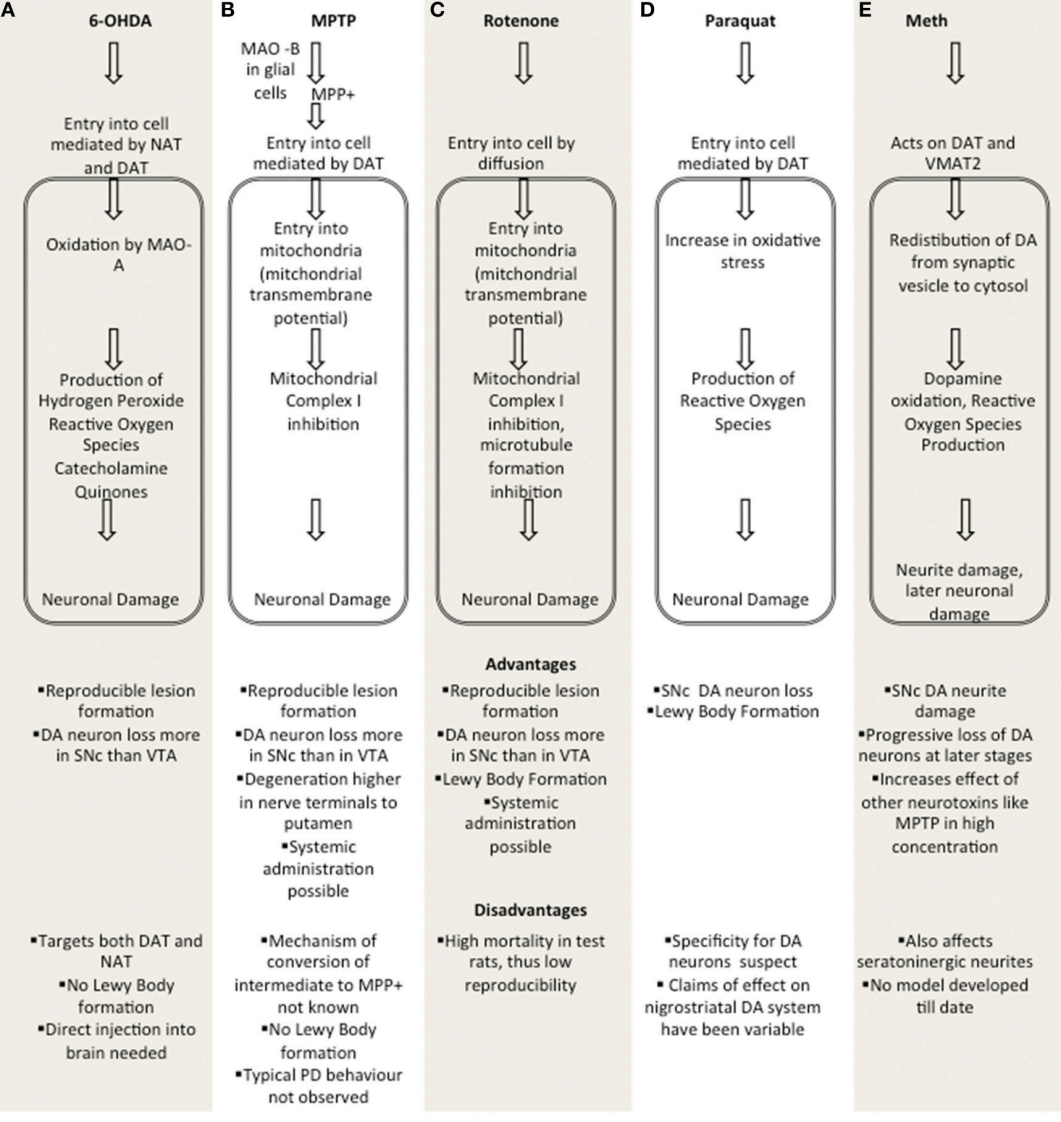
**Comparison of major toxin models**. **(A)** 6-OHDA, **(B)** MPTP, **(C)** Rotenone, **(D)** Paraquat, **(E)** Methamphetamine. MAO A, Monoamine oxidase A; MAO B, Monamine Oxidase B; DAT, Dopaminergic Transporter; NAT, Noradrenergic Transporter; SNc, Substantia Nigra pars Compacta; VTA, Ventral tegmental area; VMAT2, Vesicular Monoamine Transporter 2.

These are based on neurotoxins which allow for testing of nigrostrial DA neuron degeneration. Figure 1 summarizes major toxin models.

## 6-OHDA

6-hydroxydopamine (6-OHDA), presented in Figure 1A. is a selective neurotoxin that was first reported to cause lesions in nigrostriatal DA neurons in rats (Ungerstedt, 1968) but has been subsequently shown to work in other animals such as mice (da Conceição et al., 2010; Thiele et al., 2012). 6-OHDA accumulates in the cytosol and promotes formation of hydrogen peroxide, other reactive oxygen species and quinines by auto-oxidation (Cohen, 1984; Simola et al., 2007). Down regulation of dopamine synthesis in the lesioned striatum has also been observed with the non-lesioned striatum compensating for this by increased dopamine production (Del-Bel et al., 2014) 6-OHDA being hydrophilic cannot cross the blood brain barrier and thus administration is carried out by direct injection in the Substantia Nigra pars Compacta (SNc), Medial Forebrain Bundle (MFB) or striatum, depending on the rate at which lesion formation is desired. Injection into the SNc, MFB causes DA neuronal death in less than 24 h (Jeon et al., 1995). Striatal injection causes death of DA neurons over the course of 1–3 weeks. Injection of 6-OHDA causes progressive retrograde neuronal degeneration in the SNc and Ventral Tegmental Area (VTA) (Sauer and Oertel, 1994; Przedborski et al., 1995). In addition to DA transporters, it also targets noradrenergic transporters (Luthman et al., 1989). Thus, in addition to inducing PD symptoms, 6-OHDA also causes damage to other parts of the brain. The other disadvantage of 6-OHDA is that production of LB-like inclusions is not seen (Dauer and Przedborski, 2003). Behavioral wise, unilaterally lesioned rodents show drug induced rotational behavior (Blandini et al., 2008). Motor impairments are also observed, primarily due to impairment of limbs contralateral to the hemisphere in which the 6-OHDA is administered (Simola et al., 2007).

## MPTP

1-methyl-4-phenyl-1,2,3,6-tetrahydropyridine (MPTP), presented in Figure 1B. is a neurotoxin precursor of 1-methyl-4-phenylpyridinium (MPP+), which causes damage to the nigrostriatal DA pathway with a significant loss of DA neurons in the striatum and SNc, more similar to that seen in PD. MPTP susceptibility increases with age (Ovadia et al., 1995). MPTP is converted to an intermediate 1-methyl-4-phenyl-2,3-dihydropyridinium by the action of monoamine oxidase B in glial cells. This intermediate is then oxidized to MPP+ (Jackson-Lewis and Przedborski, 2007). MPP+ has a high affinity for the plasma membrane dopamine transporter with comparatively lower affinities for the norepinephrine and serotonin transporters (Javitch et al., 1985). Once inside dopaminergic neurons, MPP+ can be sequestrated into synaptosomal vesicles or be concentrated within the mitochondria (Ramsay and Singer, 1986) utilizing mitochondrial transmembrane potential. In the mitochondria, MPP+ blocks the electron transport chain by inhibiting Mitochondrial Complex I (Varastet et al., 1994). Due to rapid conversion of MPTP to MPP+, most chronic treatments are actually serial acute insults (Jackson-Lewis and Przedborski, 2007). Thus, a true chronic model would require continuous delivery of MPTP using devices such as osmotic pumps (Fornai et al., 2005). Rats have proven to be resistant to MPTP induced toxicity (Riachi et al., 1990). The reason for this resistance has been speculated to be because of differential MPP+ sequestration (Schmidt and Ferger, 2001). While MPTP produces the best results when used in monkeys including formation of LB like inclusions (Kowall et al., 2000) taking into account practical considerations, the MPTP mouse model is more popular. MPTP causes greater damage to DA neurons in the SNc than in VTA (Blesa et al., 2011, 2012). Recent dopaminergic neuron characterisation has found a specific DA subtype in the SNc is more vulnerable to MPTP (Poulin et al., 2014). Also the degeneration of nerve terminals to the putamen is higher than those to the caudate nucleus (Blesa et al., 2010). This too resembles PD phenotype.

Mice treated with MPTP also do not show behavior typical of PD, however alterations in motor movement are observed, where significant dopaminergic neuron loss is present (Jackson-Lewis and Przedborski, 2007). This model also has a significant weakness of the lack of formation of LBs in mice. Care must be taken to study the interaction of the drug tested with MPP+ before conclusions about its efficacy are drawn as some drugs might reduce the oxidative stress induced by MPTP. Also the conversion of MPTP to MPP+ includes an intermediate, which is oxidized to MPP+, thus an antioxidant treatment protocol might give good results by targeting this step without actually preventing DA neuron loss but this remains to be tested. Use of probenicid to competitively inhibit renal excretion of MPTP has also been shown to increase SNc neuronal loss (Meredith et al., 2002). MPTP has a big advantage because of its lipophylic nature as it can cross the blood brain barrier, thus allowing greater ease in administration, including systemic administration.

## Rotenone

Rotenone, presented in Figure 1C. occurs naturally in several plants and has been used as a broad spectrum insecticide, and pesticide. It functions by blocking the mitochondrial electron transport chain through inhibition of complex I, as seen in MPTP. Rotenone also blocks mitosis and inhibits cell proliferation. This is by perturbation of microtubule assembly and decreasing the GTP hydrolysis rate (Srivastava and Panda, 2007). Chronic systemic exposure to rotenone in rats causes many features of PD, including nigrostriatal DA degeneration. This model has been shown to reproduce almost all the features of PD, including the formation of intracellular inclusions that resemble LB (Sherer et al., 2003). Rotenone can be injected intraperitoneally, intravenously or subcutaneously for systemic treatment. It has also been directly injected into the brain stereotaxically (Xiong et al., 2009). However, despite demonstrating the slow and specific loss of DA neurons, this model is difficult to replicate due to the high mortality observed in rats, when treated with rotenone (Fleming et al., 2004). Rotenone is highly lipophilic and easily crosses the blood brain barrier (Talpade et al., 2000).

## Paraquat

N,N′-dimethyl-4,4′-bipyridinium dichloride (Paraquat), presented in Figure 1D. is one of the most widely used herbicides. It shares structural similarity to MPP+. Parquat causes oxidative stress in the cell through generation of reactive oxygen species. It has been shown to cause SNc DA neuron degeneration and like rotenone also induces formation of LB in DA neurons in mice and rats (McCormack et al., 2002; Cicchetti et al., 2005). However, large variability has been observed in cell death and specificity for DA neurons including some contradictions, with some researchers claiming that Paraquat does not cause changes in the nigrostriatal DA system (Miller, 2007). Paraquat has been used in conjuction with 2-(dithiocarboxy)aminoethylcarbamodithioato(2-)-kS, kS' manganese also called Maneb, a fungicide, which has been shown to potentiate the effects of both MPTP and Paraquat. Maneb on its own has also been shown to decrease locomotor activity and produce SNc neurons loss (Thrash et al., 2007).

## Amphetamine based models

Treatment of rodents and primates with high doses of methamphetamine has shown selective DA, serotonergic nerve terminal as well as SNc neuronal loss (Wagner et al., 1979; Thrash et al., 2009). Methamphetamine, presented in Figure 1E. causes this damage by promoting change in the distribution of DA from the synaptic vesicle to the cytosol (Howard et al., 2011). To do so, it interacts with both the dopamine transporter (DAT) and the vesicular monoamine transporter (VMAT2), resulting in promoting the collapse of vesicular proton gradients. This leads to DA oxidation within the neuronal cytosol, which causes oxidative stress within the cell by generation of hydroxyl and superoxide Reactive Oxygen Species (ROS) (Larsen et al., 2002; Cadet et al., 2007). Subtoxic concentrations of methamphetamine has been shown to protect DA neurons cells against 6-OHDA toxicity, whereas higher concentrations of methamphetamine exacerbated it (El Ayadi and Zigmond, 2011). On the other hand, despite affecting mainly the serotonergic system, MDMA can also affect DA neurons, with the repeated administration of MDMA producing degeneration of DA terminals in the striatum, and neuronal loss in the SNc (Granado et al., 2008a,b). Development of an amphetamine model of PD, especially in conjunction with other neurotoxins such as MPTP, and Paraquat might be useful.

## Genetic models

In theory genetic model of a simple disease or a syndrome can be made of a mutant gene involved in the progression of the disease in patients or even a gene that might not be validated to be involved in patients but can recapitulate some key features of the disease in the model system. The goal of making genetic model is 3-fold:

1. Understand the signaling and pathways associated with known causal gene.
2. Understand disease signaling by introducing a perturbation in signaling through a gene not found to be causal in patients but can mimic key disease and equally importantly disease like phenotypes.
3. To enable therapeutic screens.

Five genes are frequently targeted as disease models for PD and they have all been known to have causal connection in familial PD. One of the largest genome wide analysis studies for PD to date has implicated 28 independent variants across 24 loci (Nalls et al., 2014). What is not obvious is whether the genes encoded (Nalls et al., 2014) within these loci function as the main drivers, carriers or helpers of disease progression. In more common forms of PD, several gene functions are likely to be altered, hence monogenic models are expected to be less successful than toxin-induced models. That said genetic models have been of use in modeling familial PD and also have shed some light on more common PD mechanisms. Some of the model organisms have been invertebrates and one might be led to question the utility of invertebrate models that do not have SNc. While invertebrate models mimic more simplistic features such as loss of DA neurons, they provide a good vehicle to understand the genetic network, molecular signaling, and provide for first round of screening that can be followed up with further work in mammalian models. Tables 1–4 describe the various common rodent genetic models for PD, while Table 5 details fruit fly models. Some of the models are described below:

**Table 1:** **α-synuclein models in mice**.

**α-synuclein in mouse model**					
**Types**	**Promoter**	**SN neuron loss**	**Inclusion bodies**	**Motor impairment**	**References**
WT	PDGF-β	−	+	+	Masliah, 2000
WT/A53T	Thy1	−	+	+	van der Putten et al., 2000
WT/A30P/A53T	TH	−	−	ND	Matsuoka et al., 2001
WT	Prp	−	−	−	Giasson et al., 2002
A53T	Prp	−	+	+	Giasson et al., 2002
WT	Prp	−	−	−	Lee et al., 2002
A30P	Prp	−	+	−	Lee et al., 2002
A53T	Prp	−	+	+	Lee et al., 2002
WT	TH	−	−	−	Richfield et al., 2002
A53T and A30P	TH	+	−	+	Thiruchelvam et al., 2004
A30P	PrP	−	+	+	Gomez-Isla et al., 2003
WT/A30P/A53T	CMV	+	+	+	Lauwers et al., 2003
WT (1–120)	TH	−	+	+	Tofaris et al., 2006
WT	CMV	+	−	ND	St Martin et al., 2007
WT	CaMKII	+	−	+	Nuber et al., 2008
WT (1–130)	TH	+	−	+	Wakamatsu et al., 2008
WT (1–119)	ROSA26	−	−	ND	Daher et al., 2009
A53T	ROSA26	−	−	ND	Daher et al., 2009
E46K	ROSA26	−	−	ND	Daher et al., 2009
A53T	Pitx3	+	−	+	Lin et al., 2012
WT/A53T	CMVE- Syn 1	+	+	+	Oliveras-Salvá et al., 2013

*WT, Human α-synuclein; PDGF-β, Platelet Derived Growth Factor-β; Thy-1, Thy-1 Cell Surface Antigen; TH, Tyrosine Hydroxylase; Prp, Prion protein promoter; CMV, cytomegalovirus; CaMKII, Calcium/calmodulin-dependent protein kinase II; ROSA26, ROSAβgeo26P locus; Pitx3, Paired-Like Homeodomain 3; CMVE- Syn 1, cytomegalovirus enhanced synapsin 1; ND, No Data*.

**Table 2:** **LRRK2 models in mice**.

**LRRK2 in mouse model**					
**Types**	**Promoter**	**SN neuron loss**	**Inclusion bodies**	**Motor impairment**	**References**
R1441C/G^*^	mLRRK2	ND	ND	ND	Li et al., 2007
R1441C^*^	mLRRK2	−	−	−	Tong et al., 2009
WT^#^	mLRRK2	−	−	+	Li et al., 2009b
R1441G^#^	mLRRK2	−	−	+	Li et al., 2009b
WT^#^	hLRRK2	−	−	−	Melrose et al., 2010
G2019S^#^	hLRRK2	−	−	−	Melrose et al., 2010
exon 1^*^	mLRRK2	−	+	ND	Tong et al., 2010
exon 29, 30^*^	mLRRK2	−	+	ND	Tong et al., 2010
G2019S^#^	CMVE- PDGFβ	+ (DA loss)	−	−	Ramonet et al., 2011
R1441C^#^	CMVE-PDGFβ	−	−	+	Ramonet et al., 2011
WT^#^	CMVE-PDGFβ	−	−	−	Ramonet et al., 2011
G2019S^#^	CMVE-PDGFβ	+ (DA loss)	−	+	Chen et al., 2012
R1441C^#^	ROSA26	−	−	−	Tsika et al., 2014

mLRRK2, murine LRRK2; hLRRK2, human LRRK2;

* *mouse paralog*,

# human; WT, Wild Type;

*CMVE-PDGFβ, cytomegalovirus enhanced-platelet derived growth factor- β; ROSA26, ROSAβgeo26P locus; ND, No Data*.

**Table 3:** **Parkin, PINK1, and DJ-1 mice models**.

**Mouse model**					
	**Promoter**	**SN neuron loss**	**Inclusion bodies**	**Motor impairment**	**References**
**PARKIN MOUSE PARALOG**					
Exon 3 deletion	−	−	−	−	Goldberg et al., 2003
Exon 3 deletion	−	−	−	+	Itier et al., 2003
Exon 2 deletion	−	−	−	−	Perez and Hastings, 2004
Exon 7 deletion	−	−	−	−	Von Coelln et al., 2004
Exon 3 deletion	−	ND	ND	ND	Palacino et al., 2004
Truncated, Q311X	*Slc6a3*	+ (DA loss)	−	+	Lu et al., 2009
WT	nse	−	ND	ND	Bian et al., 2012
**PINK-1 MOUSE PARALOG**					
4–7 Exon mutation	−	−	−	−	Kitada et al., 2007
**DJ-1 MOUSE PARALOG**					
Exon 2 deletion	−	−	−	+	Goldberg et al., 2005,
Exon 2 deletion	−	−	ND	ND	Yamaguchi and Shen, 2007,
Exon 3–5 deletion	−	−	ND	−	Kim et al., 2005
Exon 7 inactivation	−	−	ND	+	Manning-Boğ et al., 2007
Exon 2–3 deletion	−	−	ND	−	Andres-Mateos et al., 2007
Exon 2 deletion	−	−	−	+	Chandran et al., 2008
Exon 1 stop	−	+	ND	−	Rousseaux et al., 2012

*WT, Wild Type; Slca6a3, Solute carrier family 6a3; nse, neuron specific enolase; ND, No Data*.

**Table 4:** **Genetic models in rats**.

**Rat model**					
**Types**	**Promoter**	**SN neuron loss**	**Inclusion bodies**	**Motor impairment**	**References**
**HUMAN α-SYNUCLEIN**					
A30P	BA	+ (DA loss)	+	ND	Klein et al., 2002
WT	CBA	+	+	+	Kirik et al., 2002
A53T	CBA	+	+	+	Kirik et al., 2002
WT/A30P/A53T	PGK	+ (DA loss)	+	ND	Lo Bianco et al., 2002
WT	CMV	+ (DA loss)	−	ND	Yamada et al., 2004
WT, S129D, S129A	CMV	+ (DA loss)	+	ND	McFarland et al., 2009
A53T	CBA	+ (DA loss)	+	ND	Koprich et al., 2010
WT, S87A	ND	+ (DA loss)	+	+	Oueslati et al., 2012
S87E	ND	−	+	−	Oueslati et al., 2012
WT	SYN 1	+ (DA loss)	+	+	Engeln et al., 2013
**HUMAN LRRK2**					
WT	SYN 1	−	−	ND	Dusonchet et al., 2011
G2019S	SYN 1	+	−	ND	Dusonchet et al., 2011
**PARKIN RAT HOMOLOG**					
WT	PGK	−	ND	ND	Liu et al., 2013
WT (KO -Exon 4)	−	−	−	−	Dave et al., 2014
**DJ-1 RAT HOMOLOG**					
WT (KO -Exon 5)	−	+ (DA loss)	−	+	Dave et al., 2014
**PINK-1 RAT HOMOLOG**					
WT (KO -Exon 4)	−	+ (DA loss)	−	+	Dave et al., 2014

*WT, Wild –Type; BA, beta actin; CBA, chicken beta actin; PGK, phosphoglycerate kinase; CMV, cytomegalovirus; SYN-1, synapsin I; ND, No Data*.

**Table 5:** **Genetic models in fruit flies**.

**Drosophila model**					
**Types**	**Driver**	**DA neuron loss**	**Inclusion bodies**	**Motor impairment**	**References**
**Human α-synuclein**					
WT/A30P/A53T	elav-GAL 4	+	+	+	Feany and Bender, 2000
WT/A30P/A53T	elav-GAL 4	+	+	ND	Auluck et al., 2002
WT	elav-GAL 4	−	+	+	Pesah et al., 2005
S129D	elav-GAL 4	+	+	ND	Chen and Feany, 2005
S129A	elav-GAL 4	−	+	ND	Chen and Feany, 2005
WT 71–82 removed	elav-GAL 4	−	−	ND	Periquet et al., 2007
WT 1–120 trunc.	elav-GAL 4	+	+	ND	Periquet et al., 2007
WT 1–78 trunc.	elav-GAL 4	+	+	ND	Periquet et al., 2007
**Human LRRK2**					
WT	elav-GAL 4	+	ND	+	Liu et al., 2008
G2019S	elav-GAL 4	+	ND	+	Liu et al., 2008
I2020T	elav-GAL 4	−	−	+	Venderova et al., 2009
WT	ddc-GAL4	−		−	Ng et al., 2009
G2019S	ddc-GAL4	+		+	Ng et al., 2009
Y1699C	ddc-GAL4	+		+	Ng et al., 2009
G2385R	ddc-GAL4	+		−	Ng et al., 2009
**PARKIN FLY HOMOLOG**					
p25 insertion (null)	−	−	ND	+	Greene et al., 2003
P21 insertion (null)	−	−	−	+	Pesah et al., 2004
Q311X/T240R	ddc-GAL4	+	−	+	Sang et al., 2007
**DJ-1 FLY HOMOLOG**					
DJ-1β part deletion	−	−	−	+	Park et al., 2005
DJ-1α null	−	−	−	ND	Meulener et al., 2005
DJ-1β null	−	−	−	ND	Meulener et al., 2005
DJ-1β null	−	−	ND	ND	Menzies et al., 2005
DJ-1α RNAI	−	+	ND	−	Lavara-Culebras and Paricio, 2007
DJ-1β null	−	−	ND	−	Lavara-Culebras and Paricio, 2007
**PINK-1 FLY HOMOLOG**					
Kinase domain	−	−	ND	+	Clark et al., 2006
UTR + part of exon 1	−	+	ND	+	Park et al., 2006

*WT, Wild-type; elav, Embryonic Lethal—Abnormal Vision; ddc, dopa decarboxylase; RNAI, RNA Interference; UTR, Untranslated Region; ND, No Data*.

## α-Synuclein

This gene is linked to a dominant type of familial PD and the α-synuclein protein is a major part of LBs observed in the brains of PD patients (Iwatsubo, 2003). Table 1 catalogs α-synuclein mice models, while Table 4 has rat genetic models, including α-synuclein. Mutations in five locations have so far been identified in familial PD (Polymeropoulos et al., 1997; Krüger et al., 1998; Singleton et al., 2003; Chartier-Harlin et al., 2004; Zarranz et al., 2004; Appel-Cresswell et al., 2013; Kiely et al., 2013; Proukakis et al., 2013). Injection of wild type or mutant α-synucelin protein has been shown to induce loss of DA neurons, and cause motor impairment in both mice and rats (Oliveras-Salvá et al., 2013). Several mutant lines have been developed in mice that show decreases in striatal DA, exhibit inclusion bodies, and show motor impairmentsbut several fail to show significant degeneration of nigrostrial PD neurons (Masliah, 2000; van der Putten et al., 2000; Giasson et al., 2002; Lee et al., 2002; Richfield et al., 2002; Gomez-Isla et al., 2003; Fernagut and Chesselet, 2004; Thiruchelvam et al., 2004; Tofaris et al., 2006; St Martin et al., 2007; Nuber et al., 2008; Wakamatsu et al., 2008; Daher et al., 2009). Recent use of the Pitx3 promoter shows promise as the line shows progressive SNc DA neuronal loss too along with decrease in DA release and significant motor defects (Li et al., 2009a; Lin et al., 2012). Viral Vectors such as Lentiviruses, Adeno-associated Viruses have been directly injected into the brain at the SNc near the cell bodies of DA neurons in both mice and rats (Lauwers et al., 2003, 2007). Mutant lines with DA loss and inclusion bodies have also been developed in rats (Klein et al., 2002; Lo Bianco et al., 2002; Yamada et al., 2004; McFarland et al., 2009; Koprich et al., 2010; Oueslati et al., 2012; Engeln et al., 2013). While so many models have been developed with α-synuclein, its exact function is not known. Available data suggests that α-synuclein might be a presynaptic regulator of DA release, synthesis or storage, and has been shown to be a regulator of paired-stimulus depression (PSD) (Maries et al., 2003). It also seems to play a role in neuroprotection (Quilty et al., 2006).

## LRRK 2

Mutations to this gene are known to cause an autosomal familial form of PD (Funayama et al., 2002; Paisán-Ruíz et al., 2004). Mice LRRK2 lines are compared in Table 2 and rat lines in Table 4. Mitochondrial dysfunction enhances LRRK2 neurodegeneration in some models through unclear mechanisms (Winklhofer and Haass, 2010). LRRK 2 knockout mice have been demonstrated to show abnormal aggregation and accumulation of proteins including α-synuclein, while otherwise not showing any nigrostriatal degeneration (Li et al., 2007, 2009b; Melrose et al., 2010; Tong et al., 2010; Ramonet et al., 2011; Hinkle et al., 2012; Tsika et al., 2014). Virus based models have so far shown some nigrostriatal degeneration however only partial PD phenotypes have so far been developed (Dusonchet et al., 2011; Chen et al., 2012). The LRRK2 gene codes for a 2527 amino acid long protein with multiple domains (Anand and Braithwaite, 2009). Of these domains, two enzymatic domains, the kinase domain and the GTPase domain are of particular interest. In addition multiple protein-protein interaction regions suggest that LRRK may have a role as a major signaling complex (Marín, 2006; Mata et al., 2006). More information on LRRK 2 interactions is needed.

## Parkin

Parkin mutations have been seen in cases of familial PD. Table 3 covers Parkin mice strains. Parkin is an integral ligase in the ubiquitin proteosome system (Lücking et al., 2000). Most Parkin transgenic rodents do not exhibit loss of DA neurons in the SNc (Goldberg et al., 2003; Itier et al., 2003; Palacino et al., 2004; Von Coelln et al., 2004; Perez and Palmiter, 2005; Lu et al., 2009; Bian et al., 2012; Liu et al., 2013). Some recent transgenic rodent models have demonstrated modest loss of DA neurons (Kitada et al., 2009; Dave et al., 2014; Van Rompuy et al., 2014). Popularadoption of these models awaits successful reproduction of the results.

## DJ-1

DJ-1 is molecular chaperone that under redox reductions plays a role in inhibition of α-synuclein aggregate formation (Shendelman et al., 2004). DJ-1 mutations are linked to autosomal recessive, early onset PD and genetic models using DJ-1 are cataloged in Table 3. Rat model of DJ-1 is presented in Table 4. KO models of DJ-1 show decreased DA release in the striatum but no loss of SNc DA neurons (Goldberg et al., 2005; Andres-Mateos et al., 2007; Manning-Boğ et al., 2007; Yamaguchi and Shen, 2007; Chandran et al., 2008). Hypersensitivity to neurotoxins, such as MPTP, was also observed in DJ-1 deficient mice (Kim et al., 2005). One new model, the DJ1-C57 mouse, shows promise with dramatic unilateral loss of dopaminergic (DA) neurons in the SNc that progresses to bilateral degeneration of the nigrostriatal axis with aging and mild motor behavior deficits (Rousseaux et al., 2012). If reproduced, this model would be highly beneficial to study early onset PD. A transgenic rat model of DJ-1 has also been produced, which exhibits dopaminergic neuron loss and motor abnormalities (Dave et al., 2014).

## PINK1

Mutations in the PARK6 locus of PINK1 cause a form of early-onset autosomal PD. Table 3 presents mice model of PINK1 and Table 4 rat models. PINK1 codes for a mitochondrial kinase, which recruits Parkin from the cytosol to the mitochondria, increases the ubiquitination activity of Parkin, and induces Parkin-mediated mitophagy (Lazarou et al., 2013). Since PINK1 and the Parkin function in the same pathway, the phenotypes of PINK1 and Parkin KO mice are very similar. No significant DA neuron abnormalities or LB formation have been observed in PINK1 KO mice however mitochondrial functional defects and increased sensitivity to oxidative stress were observed (Kim et al., 2005; Kitada et al., 2007). Increased levels of α-synuclein through overexpression in PINK1 KO mice results in DA loss but no degeneration in the SNc (Oliveras-Salvá et al., 2013). PINK1 KO rats exhibiting DA loss and motor impairment have been developed recently which more closely mimics PD phenotype (Dave et al., 2014).

## Non-mammalian genetic models of PD

Barring α-synuclein, most familial PD genes have at least one drosophila homolog. This includes homologs of PINK1, Parkin, DJ-1, and LRKK2 that have been presented in Table 5. Models with human α-synuclein and LRRK2 have also been developed (Feany and Bender, 2000; Auluck et al., 2002; Chen and Feany, 2005; Pesah et al., 2005; Periquet et al., 2007; Liu et al., 2008; Ng et al., 2009). These transgenic flies show some of the traits of familial PD, with well characterized loss of dopaminergic neurons and motor impairment, except in the case of DJ-1 in which only motor impairment has been observed in DJ-1 β partial deletion (Greene et al., 2003; Pesah et al., 2004; Chen and Feany, 2005; Meulener et al., 2005; Park et al., 2005, 2006; Clark et al., 2006; Lavara-Culebras and Paricio, 2007; Sang et al., 2007) *D. melanogaster* transgenic models have also helped in elucidating the role of DJ-1, Parkin and PINK1 in mitochondrial physiology (Venderova et al., 2009; Cookson, 2012). Further the study of interactions of human α-synuclein, LRRK2, Parkin, PINK1, and DJ-1 genes has also been possible in the drosophila system (Hirth, 2010).

Like *D. melanogaster, D. rerio* homologs of most familial PD genes have been discovered. Unlike the rodent and drosophila genetic models of PD, comparatively less characterisation has been carried out in *D. rerio*. Expression of human α-synuclein and knockouts of Parkin, PINK1, DJ-1 and LRRK2 have been generated, which show some success in mimicking symptoms of familial PD (Park et al., 2006; Bretaud et al., 2007; Anichtchik et al., 2008; Flinn et al., 2009; Fett et al., 2010; Sheng et al., 2010; Milanese et al., 2012; Priyadarshini et al., 2013; O'Donnell et al., 2014). Verification of the results and further behavioral testing is required to establish these models for therapeutic screens.

The advantages of transparency and complete cell lineage information make *C. elegans* an interesting model for neurodegenerative diseases. Homologs of human PD-related proteins, including Parkin, LRRK2, PINK1 and DJ-1, have been found in *C. elegans* (Springer et al., 2005; Sakaguchi-Nakashima et al., 2007; Sämann et al., 2009; Kamp et al., 2010; Lee et al., 2013; Chen et al., 2015). Transgenic models developed for these genes have shown increase sensitivity to neurotoxins like MPTP (Ved et al., 2005).

## Conclusion

Both toxin and genetic based models have their advantages and disadvantages. However, the use of the two in combination would be quite beneficial. Thus, a multi gene modulated transgenic model in combination with a reliable and effective neurotoxin might allow us to model the PD phenotype better. Addition of a miRNA or siRNA cocktail to the appropriate model systems could potentially allow for the creation of a very robust and accurate PD model showing all the symptoms of PD. Development of primary cell culture models might allow for mimicking slow development of PD cellular damage phenotype too, and be useful for drug discovery. In coming years, we expect to see better models for both basic understanding of PD and also for improved high-throughput drug-discovery.

## Author contributions

SJ conducted overall review of the field and wrote major part of this manuscript. NT co-conducted the review of the field. SS and SM provided expertise on selected topics, wrote and edited select parts of the manuscript. SK envisaged the overall study, guided the work of SJ and NT, and conducted the final editing.

## Funding

SJ was funded by CSIR Ph.D. fellowship. SK's lab is funded by intramural IISER-K funding.

### Conflict of interest statement

The authors declare that the research was conducted in the absence of any commercial or financial relationships that could be construed as a potential conflict of interest. The reviewer Dr Ines Moreno-Gonzalez and handling Editor declared their shared affiliation, and the handling Editor states that the process nevertheless met the standards of a fair and objective review.
